# Deletion of glyceraldehyde‐3‐phosphate dehydrogenase (*gapN*) in *Clostridium saccharoperbutylacetonicum N1‐4(HMT)* using CLEAVE™ increases the ATP pool and accelerates solvent production

**DOI:** 10.1111/1751-7915.13990

**Published:** 2021-12-19

**Authors:** Taylor I. Monaghan, Joseph A. Baker, Preben Krabben, E. Timothy Davies, Elizabeth R. Jenkinson, Ian B. Goodhead, Gary K. Robinson, Mark Shepherd

**Affiliations:** ^1^ School of Biosciences, RAPID Group University of Kent Canterbury CT2 7NJ UK; ^2^ Green Biologics Ltd R&D Labs 154AH Brook Drive Milton Park, Abingdon OX14 4SD UK; ^3^ School of Science, Engineering & Environment University of Salford Lancashire M5 4WT UK; ^4^ Present address: Microbesphere Ltd 9 Marsh Lane Didcot, Oxfordshire OX11 8FD UK; ^5^ Present address: Corteva Agriscience CPC2 Capital Park Fulbourne, Cambridge CB21 5XE UK; ^6^ Present address: Biocleave Limited 154AH Brook Drive, Milton Park Abingdon OX14 4SD UK

## Abstract

The development and advent of mutagenesis tools for solventogenic clostridial species in recent years has allowed for the increased refinement of industrially relevant strains. In this study we have utilised CLEAVE™, a CRISPR/Cas genome editing system developed by Green Biologics Ltd., to engineer a strain of *Clostridium saccharoperbutylacetonicum N1‐4(HMT)* with potentially useful solvents titres and energy metabolism. As one of two enzymes responsible for the conversion of glyceraldehyde‐3‐phosphate (GAP) to 3‐phosphoglyceric acid in glycolysis, it was hypothesised that deletion of *gapN* would increase ATP and NADH production that could in turn improve solvent production. Herein, whole genome sequencing has been used to evaluate CLEAVE™ and the successful knockout of *gapN*, demonstrating a clean knockout with no other detectable variations from the wild type sequence. Elevated solvent levels were detected during the first 24 h of batch fermentation, indicating an earlier shift to solventogenesis. A 2.4‐fold increase in ATP concentration was observed, and quantitation of NAD(P)H derivatives revealed a more reducing cytoplasm for the *gapN* strain. These findings expand our understanding of clostridium carbon metabolism and report a new approach to optimising biofuel production.

## Graphical abstract

This article employs a combination of genetic, genomic and microbiological techniques to demonstrate that manipulation of glyceraldehyde‐3‐phosphate activity in a solventogenic clostridium species can enhance butanol and acetone titres. These findings expand our understanding of clostridium carbon metabolism and report a new approach to optimising biofuel production.

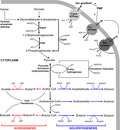

## Introduction

ABE fermentation using solventogenic clostridial species has been used for the industrial production of acetone, butanol and ethanol for over a century (for a review see Green, 2011). In the century that has passed since the discovery of the original Weizmann strain, species such as *Clostridium beijerinckii* (Jones and Keis, 1995) and *Clostridium saccharoperbutylacetonicum* (Hongo *et al*., 1969) have surpassed the original Weizmann strain as the preferred choice for ABE fermentation when grown on carbohydrate material. Unlike the well‐characterised strain *Clostridium acetobutylicum*, *C. saccharoperbutylacetonicum* N1‐4(HMT*)* is a relative newcomer to the world of industrial ABE production. First discovered in 1969 (Hongo *et al*., 1969) the full genome sequence of the strain was not published until 2014 (Poehlein *et al*., 2014). Over the years, fermentation experiments have been performed using a wide variety of feedstocks that have confirmed this strain as a butanol hyperproducer (Tashiro *et al*., 2007; Al‐Shorgani *et al*., 2012; Noguchi *et al*., 2013). Early work demonstrated that unlike *C. acetobutylicum* strains, loss of solvent production in *C. saccharoperbutylacetonicum* N1‐4(HMT*)* did not result from loss of the *sol* genes but more so from the dysregulation of the *sol* operon and upstream genes encoding other pathway enzymes (Kostan *et al*., 2010). Further to this, the mega plasmid found in *C. saccharoperbutylacetonicum* N1‐4(HMT) has also been linked to ester production of butyl acetate and butyl butyrate (Gu *et al*., 2019). To better understand butanol production in *C. saccharoperbutylacetonicum* N1‐4(HMT), the key biosynthetic genes (either endogenous or exogenous) including the *sol* operon (*bld‐ctfA‐ ctfB‐adc*), *adhE1, adhE1^D485G^, thl, thlA1^V5A^, thlA^V5A^* and the expression cassette EC (*thl‐hbd‐crt‐bcd*) were overexpressed in the strain (Wang et al., 2017b). In summary, overexpression of the *sol* operon resulted in a 400% increase in the production of ethanol with the highest increase in butanol (13.7%) seen in the strain with the over expression of the EC cassette. In an attempt to better understand and elevate the process of carbon catabolite repression (CCR), the sucrose metabolic pathway was shut down via inactivation of the gene *scrO*, resulting in a decrease in sucrose consumption by 28.9% and a decrease in ABE production by 44.1% using sucrose as the main carbon source. Additionally, deletion of the *scrR* gene alleviated CCR in the glucose/sucrose mixed fermentation, and overexpression of the endogenous sucrose pathway resulted in increased ABE production (Zhang *et al*., 2018).

Despite significant understanding of the *Clostridium* genus and its genome it is notoriously difficult to engineer. As a result, tools available for genetic engineering of *Clostridium* have lagged behind those for Gram‐negative species such as *E. coli*. Moreover, the development of tools for creation of robust strain development in *C. saccharoperbutylacetonicum* N1‐4*(HMT)* has lagged behind that of other *Clostridium* strains such as *C. acetobutylicum* and *C. beijerinckii,* where the ClosTron system developed by Heap *et al*. (2007) has been widely used (Underwood *et al*., 2009; Cartman *et al*., 2010; Cooksley *et al*., 2010, 2012; Antunes *et al*., 2011; Wietzke and Bahl, 2012; Xu *et al*., 2015). However, CRISPR/Cas technology, a powerful counter‐selection method, has recently been used to screen against homologous recombination events in Clostridium (Bruder *et al*., 2016; Huang *et al*., 2016; Nagaraju *et al*., 2016; Wang, *et al*., 2017; Wasels *et al*., 2017). Moreover, CLEAVE™ technology developed by Green Biologics has previously been shown to successfully generate strains carrying desired SNPs as well as those with deletions and insertions (Atmadjaja *et al*., 2019), although detailed whole genome sequencing (WGS) analyses have not previously been performed on the engineered strains to screen for unwanted mutations. Hence, this method was chosen for chromosomal deletions in the current study and CLEAVE™ was assessed using WGS.

**Fig. 1:**
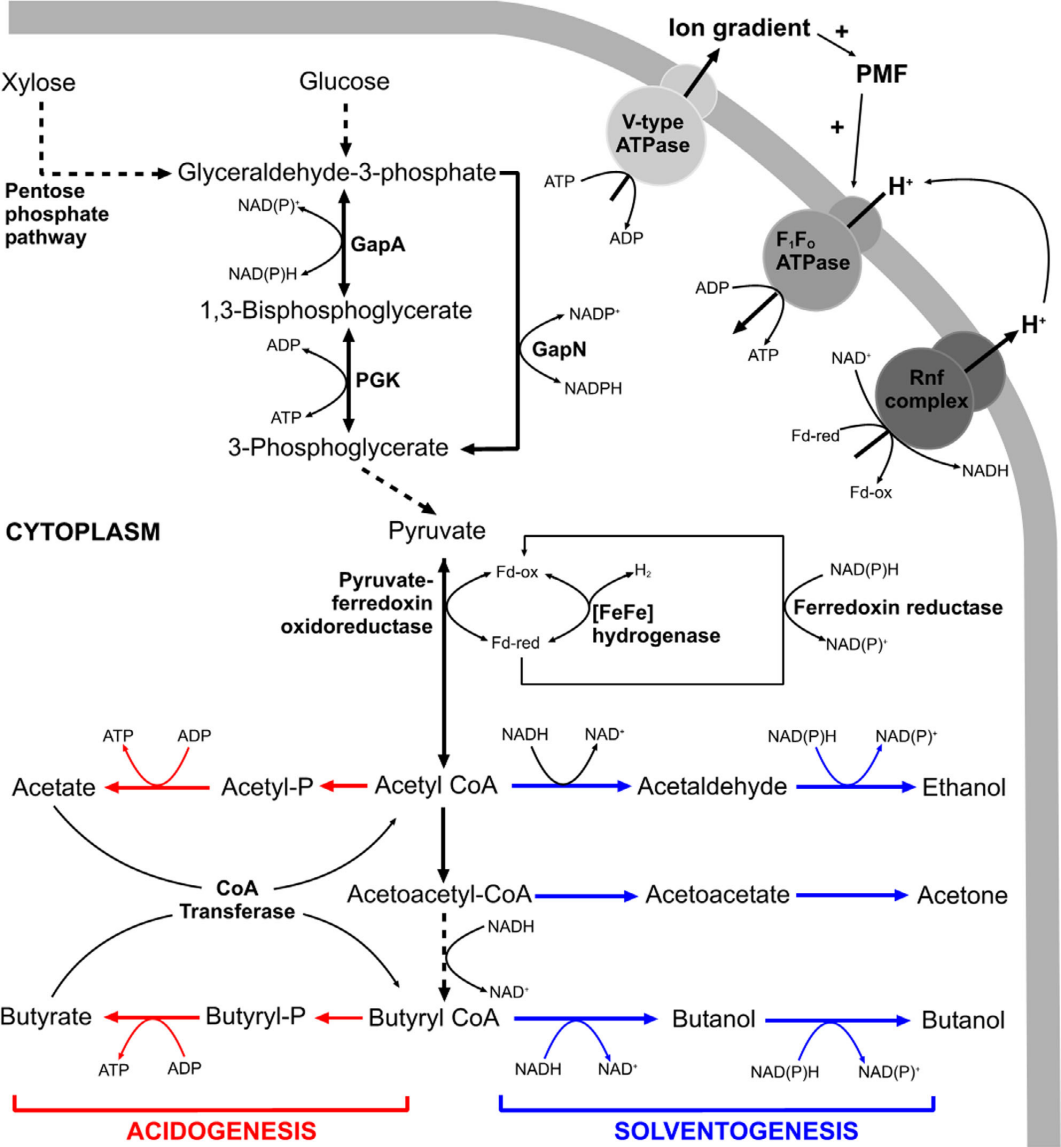
GapN is the monophosphorylating NADP‐dependant GAPDH found in *C. saccharoperbutylacetonicum N1‐4(HMT)*. Along with GapA, GapN is responsible for the conversion of glyceraldehyde‐3‐phosphate to 3‐phosphoglycerate. With the GapN reaction producing NADPH rather than NADH and also circumventing ATP generation by PGK, it is hypothesised that deletion of *gapN* will result in an increase in the concentration of ATP and elevate the NADH:NAD^+^ ratio. Pathways for acidogenesis, solventogenesis and generation of PMF are shown to highlight their involvement in consumption/production of NADH and ATP.

The enzyme of interest in the current study is GapN, a cytosolic non‐phosphorylating NADP‐dependent glyceraldehyde‐3‐phosphate dehydrogenase (GAPDH; EC.1.2.1.9) that catalyses the irreversible oxidation of glyceraldehyde‐3‐phosphate (G3P) to 3‐phospholglycerate (Fig. 1; Iddar *et al*., 2002). However, three classes of GAPDH exist that are known to be involved in the central carbon metabolism pathway, with the other two being; (i) a NAD^+^‐dependent glycolytic enzyme (EC.1.2.1.12, GapA in *C. saccharoperbutylacetonicum*) found in the cytoplasm of all organisms that plays a key role in the Embden‐Meyerhoff pathway (Fothergill‐Gilmore and Michels, 1993); and (ii) a NADP^+^‐dependent GAPDH found in photosynthetic organisms (EC. 1.2.1.13, absent in *C. saccharoperbutylacetonicum*), that is a key component of the reductive pentose‐phosphate cycle (Brinkmann *et al*., 1989). Originally discovered in photosynthetic eukaryotes (Mateos and Serrano, 1992), GapN was subsequently identified in *Streptococcus mutans* and *S. salivarius* (Boyd *et al*., 1995). Recombinant GapN from *Streptococcus mutans* has been expressed in *Corynebacterium glutamicum* as a route for NADPH generation to facilitate l‐lysine production (Takeno *et al*., 2010), and recombinant GapN from *C. acetobutylicum* has been expressed in *E. coli* and has been shown to have an absolute specificity for NADPH (Iddar *et al*., 2002). GapN activity is likely to diminish the production of NADH via GAPDH (Fig. 1), decreasing this valuable source of reducing power that is obligately required solvent production via ABE fermentation. Furthermore, GapN activity also diminishes the production of ATP by phosphoglycerate kinase (PGK; Fig. 1), which is likely to influence cell growth and potentially impact upon ABE yield. Hence, it was hypothesised that deletion of *gapN* would enhance ABE yield, presumably via increasing the production of NADH and ATP. The study herein describes the deletion of *gapN* from *C. saccharoperbutylacetonicum* using CLEAVE^TM^, assessment of this mutagenesis tool using WGS, and assessment of the impact of *gapN* deletion upon growth, acid/solvent titre, redox poise and ATP yield.

## Results

### Deletion of *gapN* in *C. saccharoperbutylacetonicum N1‐4(HMT)* using CLEAVE™

Deletion of *gapN* was carried out using the proprietary CRISPR/Cas technology CLEAVE™ developed by Green Biologics Limited (Atmadjaja *et al*., 2019). After successful construction of the pMTL82154‐*gapN*‐HR vector (Fig. S1 and Table S1) and transformation into *E. coli*, this construct was isolated and was used to transform *C. saccharoperbutylacetonicum* N1‐4*(HMT)*. Successful transformants were subjected to several rounds of sub‐culturing intended to promote a double recombination event between pMTL82154‐*gapN*‐HR and the chromosome, yielding a clean deletion of the entire *gapN* gene.

Following sub‐culturing, the cells were transformed with the killing vector pMTL83251_Ldr_DR_Sp_DR (Fig. S2) that is capable of producing crRNA that targets the PAM protospacer site derived from the middle of the *gapN* gene. The crRNA encoded by the killing vector was designed to recognize the PAM/protospacer within cells that have been unable to undergo the double recombination event and still contain the wild type *gapN* locus, resulting in killing of only these wild type cells. Following successful transformation of the killing vector, colonies were screened using the HR_F1 and HR_R2 primers that anneal upstream and downstream of *gapN* and were used in the construction of the HR vector (Fig. S1A and Table S1). Colonies that lacked *gapN* resulted in a PCR product of 1256 bp compared to the wild type at ~ 3000 bp containing the *gapN* gene. Successful colonies were sent for Sanger sequencing to confirm successful deletion of *gapN*.

### WGS of *∆gapN* and wild type *C. saccharoperbutylacetonicum N1‐4(HMT)*

To determine whether the genome editing via CLEAVE™ introduced any unwanted mutations, WGS was carried out on both wild type and ∆*gapN C. saccharoperbutylacetonicum* N1‐4*(HMT)*. Genome libraries were prepared using the Nextera XT v2 protocol and sequenced using an Illumina MiSeq benchtop sequencer according to the manufacturer’s instructions (Illumina, San Diego, CA, USA). Raw reads have been uploaded to the European Nucleotide Archive and are available under accession numbers ERS6580404 (wild type *C. saccharoperbutylacetonicum* N1‐4*(HMT)*; ‘C1’) and ERS6580405 (∆*gapN C. saccharoperbutylacetonicum* N1‐4(HMT); ‘C2’). The resulting short paired‐end reads were assessed using FastQC before and after trimming of the reads using Trimmomatic (Bolger *et al*., 2014). Trimmomatic removes adaptor sequences inserted during library preparation, as well as low quality reads and bases from each dataset. The final trimmed reads from the wild type and the *∆gapN* strain were mapped to the reference *C. saccharoperbutylacetonicum* N1‐4*(HMT)* genome obtained from Genbank (CP004121.1). The mapping was carried out using BWA and Samtools (Li, 2013), and then Qualimap (García‐Alcalde *et al*., 2012) was used to assess the quality of genome mapping of both strains (Table 1). Qualimap revealed that the coverage depth varied, with 68.6 ± 33.2 seen for the *∆gapN* strain compared to 18.8 ± 11.3 for the wild type strain. Although coverage varied, 97.12% of the total 596 655 reads for the wild type strain were mapped and 95.21% of the total 2 093 890 reads from the *∆gapN* data were successfully mapped to the reference genome.

**Table 1:** Qualimap results of ∆*gapN* and wild type *C. saccharoperbutylacetonicum* N1‐4(HMT) genome sequences following read mapping to the *C. saccharoperbutylacetonicum* N1‐4(HMT) reference genome from Genbank (CP004121.1).

Characteristic	*∆gapN*	Wild type
Reference size (bp)	6 530 257	6 530 257
Number of reads	2093 890	596 655
Mapped reads	1 993 694/95.21%	579 484/97.12%
Supplementary alignments	1509/0.07%	247/0.04%
Unmapped reads	100 196/4.79%	17 171/2.88%
Read min/max/mean length (bp)	30/251/224.9	30/251/212.68
Clipped reads	21 456/1.02%	4726/0.79%
Mapping quality	59.11	59.30
Mean coverage	68.59 ± 33.2	18.8 ± 11.3

The mapped Illumina reads for the ∆*gapN* strain were analysed for variations compared to the reference genome using Snippy (https://github.com/tseemann/snippy). All 17 variations that were identified in the *∆gapN* WGS (Table 2) were manually compared to our wild type WGS using the Integrated Genome Viewer (Robinson *et al*., 2011), and were found to be present in both our wild type parent strain and the ∆*gapN* strain. This indicates that these variations are not due to the CLEAVE^TM^ genome editing process and merely reflect minor differences in our genome sequences compared to the reference strain (CP004121.1).

**Table 2:** Nucleotide variations between assembled ∆*gapN* strain and reference strain from Genbank (CP004121.1).

Position	Gene	Product	Type	Strand	Reference	∆*gapN*
41500	***–***	***–***	del		GTTTTTG	GTTTTG
297173	*mdtN_1*	Multidrug resistance protein MdtN	ins	+	GAAGTAAA	GAAGTAAAAGTAAA
807822	***–***	***–***	snp		G	T
2136980	*ybdL*	Methionine aminotransferase	del	+	TAG	TG
2136989	*ybdL*	Methionine aminotransferase	complex	+	AAAGA	AG
2137002	*ybdL*	Methionine aminotransferase	del	+	ATTTTTTG	ATTTTTG
2169690	*01968*	hypothetical protein	snp	***–***	G	A
2170090	*01968*	hypothetical protein	snp	***–***	T	C
2170579	*01968*	hypothetical protein	snp	***–***	C	T
2170673	*01968*	hypothetical protein	del	***–***	CGCCTTGACGACCTTGAGAG	CG
2171011	*01968*	hypothetical protein	snp	***–***	C	T
3257186	*rocR_1*	Arginine utilisation regulatory protein RocR	snp	+	T	G
3506222	*03225*	Nucleotidase	snp	***–***	T	C
3651781	*rsgI_2*	Antisigma‐I factor RsgI	ins	***–***	GG	GATGGAGTTG
4705891	***–***	***–***	snp		G	A
6036488	*fdtB_2*	dTDP‐3‐amino‐3,6‐dideoxy‐alpha‐d‐galactopyranose transaminase	snp		C	T
6036553	*fdtB_2*	dTDP‐3‐amino‐3,6‐dideoxy‐alpha‐d‐galactopyranose transaminase	snp		G	A

del, deletion; ins; insertion; snp; single nucleotide polymorphism.

Comparison was performed using Snippy. The ∆*gapN* deletion is not shown.

### Solvent quantitation reveals an earlier entry to solventogenesis for the ∆*gapN* strain

**Fig. 2:**
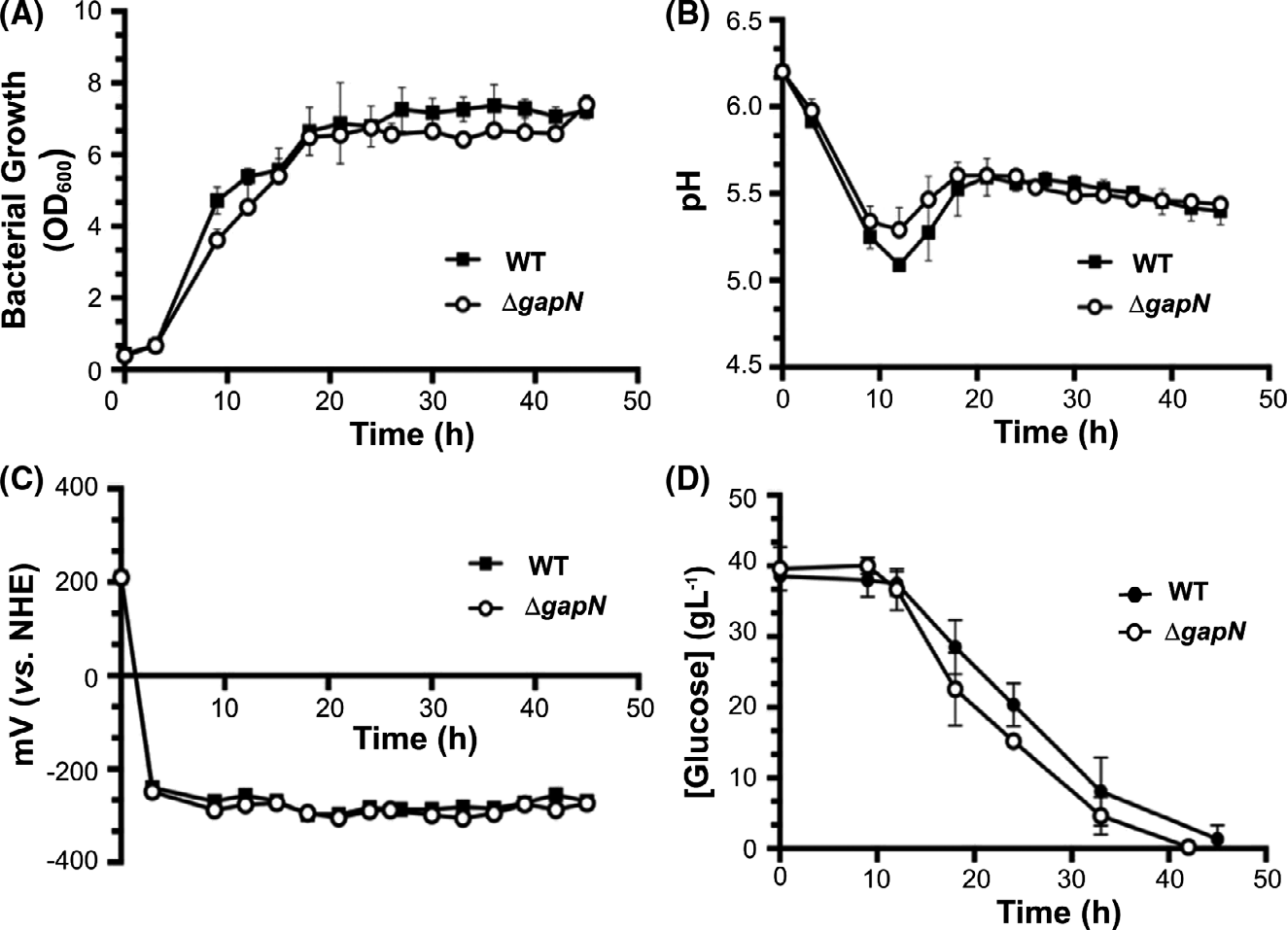
Fermentation measurements for *∆gapN* (o) and wild type (•) *C. saccharoperbutylacetonicum* N1‐4(HMT). Cultures were grown on 40 g l^−1^ glucose in YETM media in bespoke bioreactors (Monaghan *et al*., 2021); 0.1 M MES was supplemented as a pH buffering agent and the following parameters were monitored: (A) OD_600_; (B) pH; (C) redox potential of media (mV vs. NHE); (D) Glucose concentrations in growth media. Data points are mean values of a total of six repeats including three biological repeats. Error bars represent the standard deviation of mean.

The *∆gapN* and wild type *C. saccharoperbutylacetonicum* N1‐4*(HMT)* strains were cultured via batch fermentation in YETM medium containing 40 g l^−1^ glucose and supplemented with 0.1 M MES for pH control. Both strains reached similar cell densities, with maximal OD_600_ values of 7.2 ± 0.2 for the wild type and 7.4 ± 0.3 for the *∆gapN* strain (Fig. 2A). Between 9 h and 21 h in the fermentation, the media pH for *∆gapN* strain was less acidic throughout compared to the wild type *C. saccharoperbutylacetonicum* N1‐4*(HMT)* (Fig. 2B). The redox potential of the growth media for both strains behaved in a predictable manner, both dropping to ~ −300 mV vs. NHE in the first few hours (Fig. 2C), and the rate of glucose consumption was greater between 10 h and 20 h the *∆gapN* strain (Fig. 2D).

**Fig. 3:**
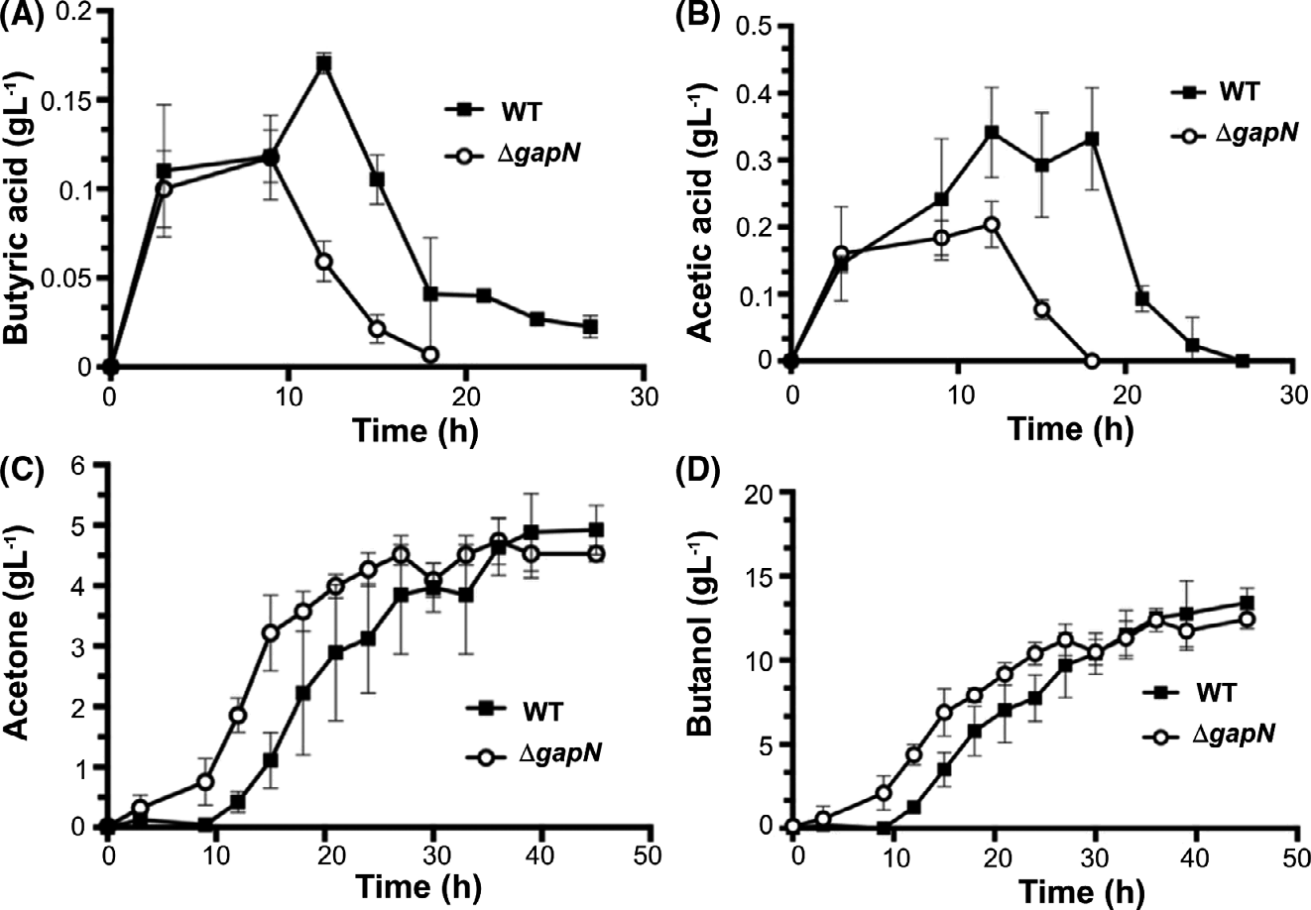
Acid and solvent profiles for ∆*gapN* (o) and wild type (•) *C. saccharoperbutylacetonicum N1‐4*(HMT) grown on 40 g l^−1^ glucose in YETM media in bespoke bioreactors (Monaghan *et al*., 2021); 0.1 M MES was supplemented as a pH buffering agent and concentrations of the following metabolites were monitored: (A) butyric acid; (B) acetic acid; (C) acetone; (D) butanol. Data points are mean values of a total of six repeats including three biological repeats. Error bars represent the standard deviation of mean.

The products of acidogenesis and solventogenesis were then analysed for the *∆gapN* and wild type strains for the growth experiments described in Fig. 2. The *∆gapN* strain exhibited reduced concentrations of acids throughout the fermentation (Fig. 3A and B). In addition, depletion of acid concentrations in the *∆gapN strain* also occurred at a faster rate in the *∆gapN* strain compared to the wild type (Fig. 3C and D). The largest observed discrepancies between the strains for acetic acid and butyric acid measurements were observed at 15 h: 0.057 g l^−1^ of butyric acid and 0.073 g l^−1^ of acetic acid were measured for the ∆*gapN* strain, compared to 0.170 g l^−1^ of butyric acid and 0.29 g l^−1^ of acetic acid for the wild type strain. The concentrations of acetone and butanol produced by the *∆gapN* strain were higher during the first 30 h of the fermentation, and the largest observed discrepancies between the strains for acetone and butanol measurements occurred at 15 h: the concentration of acetone produced by the ∆*gapN* strain was 3.2 g l^−1^ compared with only 1.1 g l^−1^ in the wild type strain; [butanol] produced by the ∆*gapN* strain was 6.87 g l^−1^ compared to 3.5 g l^−1^ in the wild type strain. Peak concentrations of butanol in the ∆*gapN*, however, were similar to those of the wild type cultures at ~ 13 g l^−1^.

Since the maximal concentrations of butanol and acetone were similar for *∆gapN* and wild type strains, it was hypothesised that solvent toxicity was the limiting factor for these measurements. To test this hypothesis, a butanol toxicity test was carried out that measured growth rates in the presence of varying concentrations of solvents. These data indicate that deletion of *gapN* does not affect the ability of *C. saccharoperbutylacetonicum* to tolerate solvent toxicity (Fig. S3).

### Deletion of *gapN* results in a reducing cytoplasm with a higher ATP pool

**Fig. 4:**
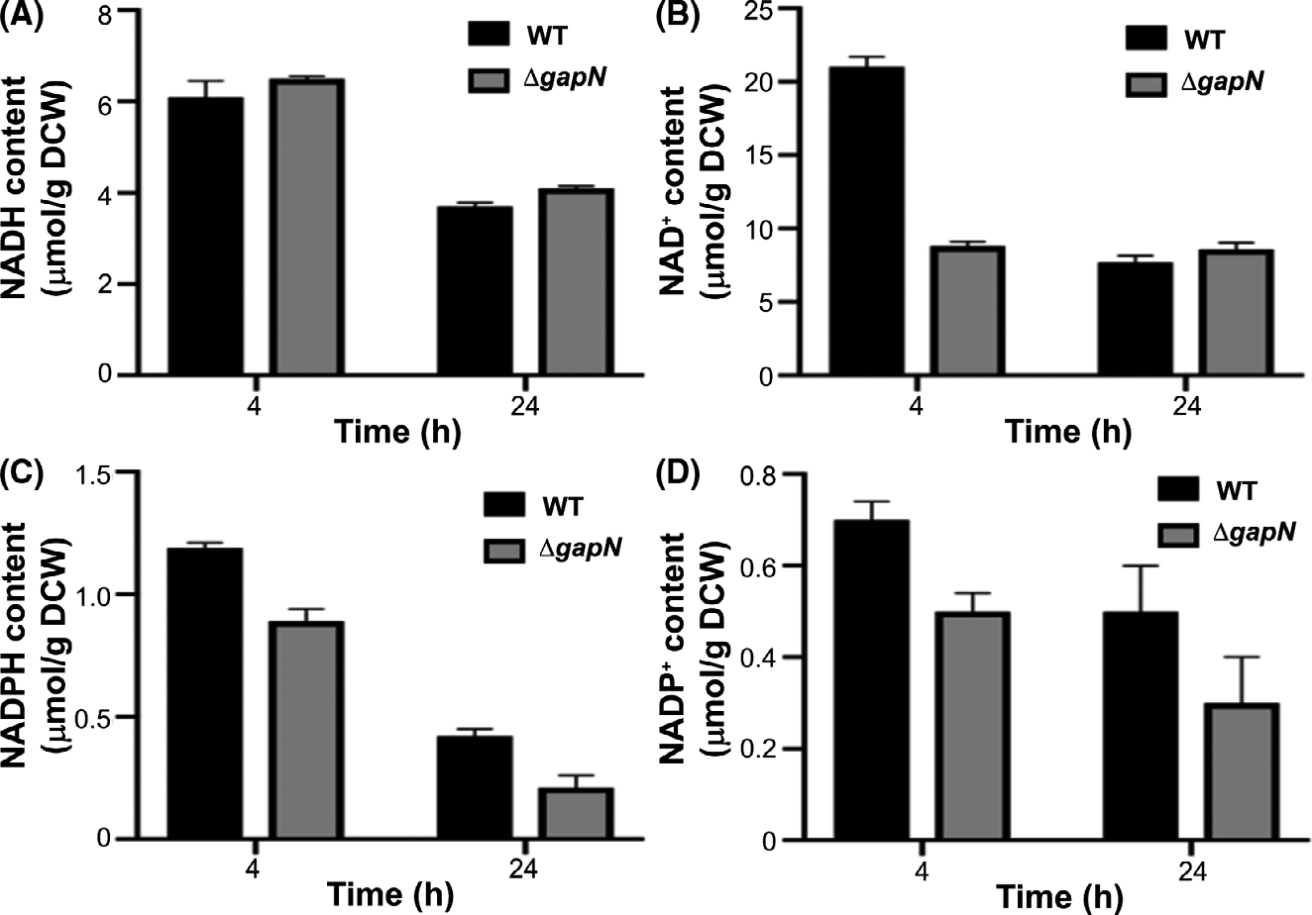
Quantitation of NAD(P)H derivatives in wild type and ∆*gapN* strains during acidogensis (4 h) and solventogenesis (24 h). Levels of NADH (A), NAD^+^ (B), NADPH (C) and NADP^+^ (D) were measured in *C. saccharoperbutylacetonicum* cells after 4 h and 24 h of growth. Cells were grown in YETM 40 g l^−1^ glucose in 50 ml serum bottles. Data points are mean values of a total of six repeats including three biological repeats. Error bars represent the standard deviation of mean.

Herein, it was hypothesised that loss of *gapN* may increase the NADH:NAD^+^ ratio, which could enhance the reducing power required for ABE fermentation (Fig. 1). Hence, it was of interest to quantify the NAD(P)H derivatives during fermentation. Due to the labile nature of NAD(P)H derivatives, cells were grown in serum bottles and assayed at 4 and 24 h to provide concentrations during early exponential phase (acidogenesis) as well as in late exponential/stationary phase (solventogenesis). Deletion of *gapN* did not appear to affect the concentrations of NADH and NADPH although a marked reduction in the concentrations of NAD^+^ and NADP^+^ during both acidogensis and solventogenesis was observed (Table S2), which is indicative of a more reducing environment in the ∆*gapN* strain compared to the wild type (Fig. 4).

**Fig. 5:**
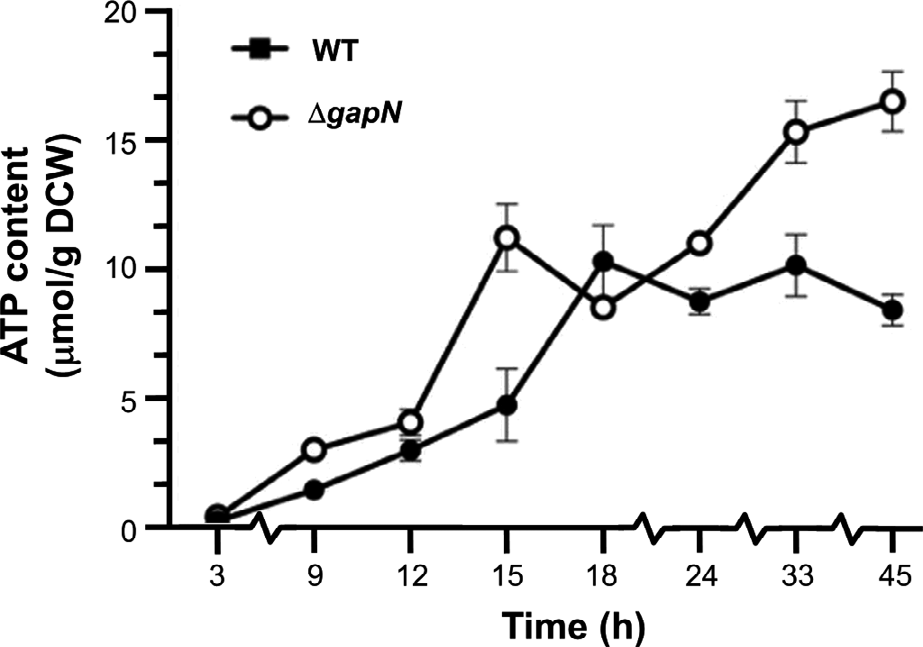
ATP measurements for ∆*gapN* (o) and wild type (•) strains grown in 40 g l^−1^ glucose in YETM media in bespoke bioreactors (Monaghan *et al*., 2021); 0.1 M MES was supplemented as a pH buffering agent. Data points are mean values of a total of six repeats including three biological repeats. Error bars represent the standard deviation of mean.

Deletion of *gapN* was also hypothesised to elevate ATP levels, so the concentrations of ATP were also monitored during fermentation in bespoke bioreactors (Monaghan *et al*., 2021) and were found to be higher in the ∆*gapN* strain compared to wild type for the majority of fermentation period (Fig. 5). The greatest difference in ATP concentrations between the ∆*gapN* and wild type strains was observed at 15 h, where 11.2 µmol g^−1^ cell dry mass was recorded for the ∆*gapN* strain compared to 4.7 µmol g^−1^ cell dry mass for the wild type.

## Discussion

The *gapN* gene was successfully deleted in *C. saccharoperbutylacetonicum* and no unwanted mutations were detected in the ∆*gapN* strain compared to the isogenic wild type parent strain, as confirmed by WGS. The ∆*gapN* strain grew at a similar rate to the wild type, with a slightly more rapid consumption of glucose during exponential phase (Fig. 2). However, the most striking difference in the ∆*gapN* strain was that acetic acid and butyric acid were converted to acetone and butanol much earlier in the fermentation (Fig. 3), reflecting an earlier shift into solventogenesis and higher solvent titres in exponential phase compared to the wild type. Ultimately, the final titre of butanol was the same as the wild type strain, probably reflecting butanol toxicity as the limiting factor. As there was no difference in butanol tolerance between the wild type and mutant strains (Fig. S3), it would be interesting to engineer butanol tolerance into ∆*gapN* and wild type strains to investigate the limits of butanol production during the early solventogenic shift.

In solventogenic *Clostridia*, acid production facilitates the generation of the majority of cellular ATP as a result of kinase activity of the enzymes involved in acetic and butyric acid production (Grupe and Gottschalk, 1992; Dürre and Hollergschwandner, 2004; Fig. 1). The associated ATP generation in acidogenesis is important as it enables the cells to produce adequate concentrations of ATP for vegetative growth, as well as to establish an ion gradient via V‐type ATPases that will enhance the electrical component of the proton motive force (PMF) that drives NADH production via the RnF complex with concomitant oxidation of reduced ferredoxin (Poehlein *et al*., 2017). This ion gradient enables ADP + Pi recycling when the cells shift into solventogensis via the use of F_1_F_O_ ATPases (Jones and Woods, 1986), as illustrated in Fig. 1. The *∆gapN* strain was shown to have elevated concentrations of ATP (compared to the wild type strain) for a sustained period during batch fermentation (Fig. 5). Between 15 and 24 h there is an observed drop in the measured ATP concentration of the ∆*gapN* strain over the wild type. This observed decrease in ATP corresponds to the time points with the lowest acid titres and the most rapid rate of solvent production in the *∆gapN* strain (Fig. 3). These data suggest that the deletion of ∆*gapN* increases the ATP concentration such that the *∆gapN* strain is able to diminish acid production and allow for an earlier shift into solventogenesis.

As well as an increase in ATP production, there is an increase in the ratio of NADH:NAD^+^ in the *∆gapN* strain. ATP production, ∆pH generated by acid production and reducing conditions within the cell play a key role in the shift to solventogenesis (Wang *et al*., 2012; Wietzke and Bahl, 2012; Zhang *et al*., 2014; Liu *et al*., 2018). Previous work on *C. acetobutylicum* reported that overexpression of both 6‐phosphofructokinase (PfkA) and pyruvate kinase (PykA) resulted in an increase in intracellular ATP and NADH, which was also accompanied by elevated butanol and ethanol production (Ventura *et al*., 2013). It has been shown that in cultures with lower ATP levels acidogenesis is a major route for energy generation, whereas cultures with elevated ATP levels produce more solvents (Meyer and Papoutsakis, 1989). Enhanced butanol production has also been observed as a result of blocking NAD(P)H consumption in *Clostridium beijerinckii* NCIMB 8052 where insertional inactivation of a NADH‐quinone oxidoreductase (*nuoG*) resulted in increased NAD(P)H and ATP as well as elevated butanol production (Liu *et al*., 2016). Overall, it can be seen that increases in both ATP and NAD(P)H in solventogenic *Clostridium* aids in maximising the solvent producing potential of the bacteria.

Previous metabolic flux analysis in in *C. acetobutylicum* (Yoo *et al*., 2015) has revealed that GapN is poorly expressed during solventogenesis, with 0.56 mRNA molecules per cell in comparison to 66 mRNA molecules per cell of GAPDH. This low expression results in only 3500 molecules of GapN protein per cell compared to 190 000 molecules of GAPDH. This study estimated that GapN would be responsible for only 5% of flux through the glyceraldehyde‐3‐phosphate oxidation pathway, so it was initially surprising that in the current study deletion of *gapN* in *C. saccharoperbutyacetonicum* elicited such significant changes in solvent production, NADH:NAD^+^ ratio and ATP yield. However, there are significant metabolic differences between *C*. *acetobutylicum* and *C. saccharoperbutyacetonicum*, including the absence of the Rnf complex in the former, that could account for variations between species.

In conclusion, we have engineered a *C. saccharoperbutylaconicum* strain that switches earlier to solventogenesis and have provided new insights into the role of GapN in controlling redox poise and ATP yield.

## Experimental procedures

### Bacterial strains, plasmids and culture conditions

The clostridium strain used in this study was Clostridium *saccharoperbutylacetonicum N1‐4(HMT)* and plasmids used were based upon pMTL82154 or pMTL83251 (Heap *et al*., 2007). Liquid cultures were recovered from 15% glycerol stocks and grown at 32°C in reinforced clostridium media (RCM) (Sigma) that had been autoclaved in sealed serum bottles. *E. coli* strains were grown aerobically at 37°C in Luria‐Bertani media supplemented with the appropriate antibiotic when required.

### Chromosomal mutagenesis

The *gapN* gene was deleted in *C. saccharoperbutylacetonicum N1‐4(HMT)* using CLEAVE as previously described (Atmadjaja *et al*., 2019), and all oligonucleotides are listed in Table S1. Firstly, a homologous recombination vector was generated with a deletion cassette that contains homology arms that are able to replace all of or part of the intended site of mutation. To construct the *gapN* deletion cassette, colony PCR of *C. saccharoperbutylacetonicum* N1‐4*(HMT)* was carried out (Fig. S1A) to amplify 2 × 1 Kbp regions upstream and downstream of *gapN*, containing complementary sequences such that it was possible to generate a seamless in‐frame deletion cassette fragment that lacked the *gapN* gene. Following successful PCR reactions, fragment isolation and Sanger sequencing to confirm the correct sequence of the deletion cassette, it was successfully blunt‐end cloned into the StuI restriction site of pMTL82154 (Fig. S1B). The new pMTL82154_*gapN*_HR vector was first transformed into *E. coli*. The HR vector pMTL82154 is a lineage of the previously described pMTL80000 shuttle vector system (Heap *et al*., 2007).

The killing vector, pMTL83251_Ldr_DR_Sp_DR, is also derived from the pMTL8000 shuttle vector system (Heap et al., 2007). The parent pMTL83251 vector contains a Gram‐positive replicon pCB102, *ermB* antibiotic marker, ColE1 + tra Gram‐negative replicon and a multiple cloning site (MCS). Pre‐engineered into the MCS of pMTL83251 is the native leader sequence (Ldr), a 181 bp sequence that is found downstream of the Cas2 machinery in *C. saccharoperbutylacetonicum* N1‐4*(HMT)* (Atmadjaja *et al*., 2019). With the leader sequence pre‐engineered into pMTL83251, it allows for the construction of CRISPR/Cas clusters on the plasmid that target the cleavage of specific chromosomal loci. The CRISPR/Cas targeting system seen in *C. saccharoperbutylacetonicum* N1‐4*(HMT)* is comprised of a target‐specific spacer flanked by direct repeats (DR_Sp_DR) that is downstream of the Cas2 sequence. For the successful deletion of *gapN,* a DR_Sp_DR cluster was designed (Fig. S2A), synthesised, and successfully cloned into pMTL83125 (Fig. S2B). The 35 bp spacer sequence that was used in the DR_Sp_DR cluster is present within the native *gapN* gene.

### Whole genome sequencing

WGS was carried out using the Illumina MiSeq system. Genome libraries for both the wild type and ∆*gapN* strains were prepared using the Nextera^®^ XT DNA Library Prep Kit, which is optimised for small genomes. Once the libraries were prepared, they were sequenced using an Illumina MiSeq benchtop sequencer. Following Illumina^®^ MiSeq, the read quality was assessed with FastQC (http://www.bioinformatics.babraham.ac.uk/projects/fastqc). To improve read quality, reads were trimmed using Trimmomatic (Bolger *et al*., 2014). Trimmomatic improves read quality of the Illumina reads by removing sequence adapters, trimming bases at the beginning and end of the reads if they fall below the stipulated threshold, followed by eliminating any of the reads that fall below the minimum read length (default setting of 36 bp). Following this, reads were mapped using BWA (Li, 2013) to the reference genome for *C. saccharoperbutylacetonicum* N1‐4(HMT) obtained from Genbank (CP004121.1). The quality of the genome maps were assessed using Qualimap (García‐Alcalde *et al*., 2012). Functional annotation was performed using PROKKA (Seemann, 2014) and Roary (Page *et al*., 2015) was used for analysis of gene content and the pangenome.

### Fermentation conditions

ABE fermentation was carried out as recently described using a bespoke anoxic bioreactor system (Monaghan *et al*., 2021). Briefly, 1 l Pyrex Quickfit culture vessels were used (SciLabware, Hartlepool, United Kingdom) with 500 ml culture volumes at 32°C and media consisted of yeast extract tryptone media (YETM) (40 g l^−1^ glucose, 2.5 g l^−1^ yeast extract, 2.5 g l^−1^ tryptone, 0.5 g l^−1^ ammonium sulphate and 0.025 g l^−1^ iron sulphate) at pH 6.2, supplemented with 0.1 M 2‐(N‐morpholino)ethanesulfonic acid (MES) free acid (Merck) for pH control. MES buffer was chosen as it has a pK_a_ of 6 and has previously been shown to be the very effective at the pH range observed during ABE fermentation. Anaerobic conditions in the fermentation were generated by sparging filtered (0.2 µm pore size) oxygen‐free nitrogen through the fermentation media for 20 min pre‐inoculation and then 5 min post‐inoculation. Seed cultures were established by growing recovered RCM grown cells, in 80 ml YETM in serum bottles overnight to an OD_600_ of ~ 4.0. The final inoculation was 10% (v/v). Fermentations were carried out in triplicate. Throughout the fermentations OD_600_, pH and redox poise (Inlab Redox Micro electrode from Mettler Toledo, Leicester, United Kingdom) of the fermentation media was measured. The redox electrode was calibrated against quinhydrone (87 mV at pH 7.0, 264 mV at pH 4.0, Normal Hydrogen Electrode (NHE) correction for a Ag/AgCl electrode in 3 M KCl = +210 mV). Following sampling, supernatant and cells were separated by centrifugation at 8000 *g* for 10 min. Supernatant and cell pellet were separated and frozen at −80°C for later use.

### Acid and solvent quantitation

Acids and solvents from culture supernatants were quantitated using Gas Chromatography Mass Spectrometry (GCMS) using an Agilent 6890N instrument as previously described (Monaghan *et al*., 2021). The GC was equipped with a Phenomenex 7HG‐6013‐11 Zebron column. Helium (> 99.999%) was used as the carrier gas, with a constant flow rate of 1 ml min^−1^. A 0.2 µl water sample was injected with a 100:1 split. Injection temperature was set to 150°C, the GCMS transfer line temperature was set to 280°C, ion source to 230°C, and quadrapole to 150°C. After injection, column temperature was held at 30°C for 5 min, after which this increased at a linear gradient to 150°C at the 20 min mark. Compounds were identified by comparing retention times of each of the compounds with retention times of reference compounds.

### Quantitation of NADH and NADPH

NADH, NADPH and their oxidised derivatives were extracted essentially as previously described (Beri *et al*., 2016). Briefly, 2 ml of bacterial culture was added to 1 ml of 1 M HCl for NAD(P)^+^ extraction and 1 ml of 1 M KCL for NAD(P)H extraction. Cells were incubated at 55°C for 10 min and then the pH was adjusted to pH 6.5 for acidic samples and pH 7.5 for basic samples using 1 M HCl and 1 M KOH dropwise with continual vortexing. Samples were centrifuged for 15 min at 4400 *g*, with the supernatant being saved for subsequent analysis. To determine the dry cell weight (DCW), cells were harvested were centrifuged at 12 000 rpm for 5 min, washed twice with distilled water, and dried to a constant weight at 80°C.

Concentrations of NADH and NADPH were measured using previously described methods (Nisselbaum and Green, 1969; Bernofsky and Swan, 1973; Baker, 2016). In brief, the reaction mixture for the NADH assay consisted of 100 µl 1 M tricine‐NaOH (pH 8), 100 µl 40 mM EDTA, 100 µl 0.1 M NaCl, 100 µl 4.2 mM MTT, 100 µl 16.6 mM PES, 100 µl 100% EtOH. The reaction mixture for NADPH assay consisted of 100 µl 1 M tricine‐NaOH (pH 8), 100 µl 40 mM EDTA, 100 µl 0.1 M NaCl, 100 µl 4.2 mM MTT, 100 µl 16.6 mM PES, 100 µl 10 mM Glucose‐6‐phosphate; 500 µl of each assay mixture was added to 100 µl of extracted sample, topped up to 900 µl with 0.1 mM NaCl and incubated at 37°C for 5 min. To generate standard curves, similar reactions were set up where the extracted sample was replaced with nucleotide solutions of known concentration; 100 µl of alcohol dehydrogenase (Sigma Aldrich, Burlington, MA, USA) and 100 µl glucose‐6‐phosphate dehydrogenase (Sigma Aldrich) were then added (10 U of each) per reaction. The reaction was incubated at 37°C for 1 h in the dark. 500 µl of 5 M NaCl was then added to stop the reaction and precipitate the MTT. Samples were centrifuged at 10 000 *g* and 4°C for 5 min. Supernatants were decanted, MTT pellets were resuspended in 1 ml of ethanol and absorbance was measured at 570 nm.

### ATP Luminescence assay

ATP was quantified in cells growing in YETM medium using a luminescence assay according to the manufacturer’s instructions (Abcam ab113849, Cambridge, United Kingdom). To determine the DCW, cells were harvested were centrifuged at 12 000 rpm for 5 min, washed twice with distilled water, and dried to a constant weight at 80°C.

### Sugar quantitation

Sugars were quantitated essentially as previously described (Monaghan *et al*., 2021). Briefly, culture supernatants were homogenised and centrifuged at 13 400 *g* for 5 min; 200 µl of the sample was then added to 600 µl of HPLC grade water, achieving a ×4 dilution and a total volume of 800 µl. Glucose concentrations were measured using cation exchange chromatography at 60°C using a Phenomenex Rezex ROA H+ column at 1 ml min^−1^ 5 mM sulphuric acid using an Agilent 1100 series refractive index detector to monitor glucose elution. Concentrations of samples were determined by comparison to a standard curve for glucose with integrated peak areas used for the determination of glucose concentration.

### Butanol toxicity

To test butanol toxicity, 30 ml serum bottles were filled with RCM, inoculated with 15% *C. saccharoperbutylacetonicum* glycerol stocks, and were grown overnight at 32°C. The overnights were then used to inoculate (10% v/v) YETM media containing 40 g l^−1^ glucose in serum bottles under anaerobic conditions. The cells were left to grown for 4 h at 32°C until an OD_600_ of ~ 1 was reached, when they were challenged with various concentrations of butanol ranging from 0.5% to 5% (v/v). Subsequent growth rates were then monitored to assess the impact of butanol toxicity.

## Conflict of interest

The authors declare no conflicts of interest.

## Author contribution

TIM contributed to experimental design, performed the experiments and wrote the paper; JAB developed the sugar analysis methodology; PK, ETD and ERJ discussed the experiments and results; IBG supervised the genome sequencing/assembly/annotation work; GKR contributed to experimental design and edited the paper; MS was responsible for overseeing experimental design and wrote the paper.

## Supporting information


**Fig. S1**. Generation of a homologous recombination vector for *gapN* deletion. A) PCR approach to generate the deletion cassette: (i) Amplification of 1 kb fragments upstream and downstream of the *gapN* gene with 48 bp of complementary sequences; (ii) Two 1 kb PCR products with complementary ends. (iii) Product of overlap‐extension PCR, ready to be blunt‐end ligated into pMTL82154. B) Vector map of ‘pMTL82154_*gapN*_HR’ that contains the homologous recombination (HR) fragment (i.e. the deletion cassette) cloned into the StuI site of pMTL82154 (verified via StuI restriction digests and sequencing). pMTL82154_*gapN*_HR contains a pBP1 Gram‐positive replicon, *catP* antibiotic maker, ColE1 +tra Gram‐negative replicon and a *catP* reporter gene.

**Fig S2**. Generation of a killing vector for elimination of transformants that do not contain the *gapN* deletion. A) Overview of the killing vector targeting cassette for endogenous CRISPR‐Cas for genome editing. The native leader sequence (Ldr) is a 181 bp sequence found downstream of the Cas2 machinery in *C. saccharoperbutylacetonicum* N1‐4(HMT) (Atmadjaja et al., 2019). The CRISPR/Cas targeting system is comprised of a target‐specific spacer (i.e. *gapN* spacer) flanked by direct repeats (DR_Sp_DR) that is downstream of the Cas2 sequence. B) Vector map of ‘pMTL83251_*L*dr_HR_Sp_HR’ that contains the targeting cassette from panel A. Successful cloning was confirmed via colony PCR and sequencing.

**Fig. S3**. Butanol toxicity test of wild type (black bars) and ∆*gapN* (grey bars) strains of *C. saccharoperbutylacetonicum* N1‐4(HMT). Cells were grown to an OD_600_ of 1 and were then challenged with varying [butanol]. Doubling times were calculated for the 48 h of growth that followed solvent addition.

**Table S1**. Oligonucleotides used in this study.
**Table S2**. Ratio of nucleotide cofactors in wild type (WT) and ∆*gapN* C. *saccharoperbutylacetonicum* N1‐4(HMT).
