# Supplementary figures and images for: Deletion of glyceraldehyde‐3‐phosphate dehydrogenase (gapN) in Clostridium saccharoperbutylacetonicum N1‐4(HMT) using CLEAVE™ increases the ATP pool and accelerates solvent production
Source: Microb Biotechnol. 2021 Dec 19;15(5):1574–85. doi: 10.1111/1751-7915.13990 (PMC9049615; doi:10.1111/1751-7915.13990)

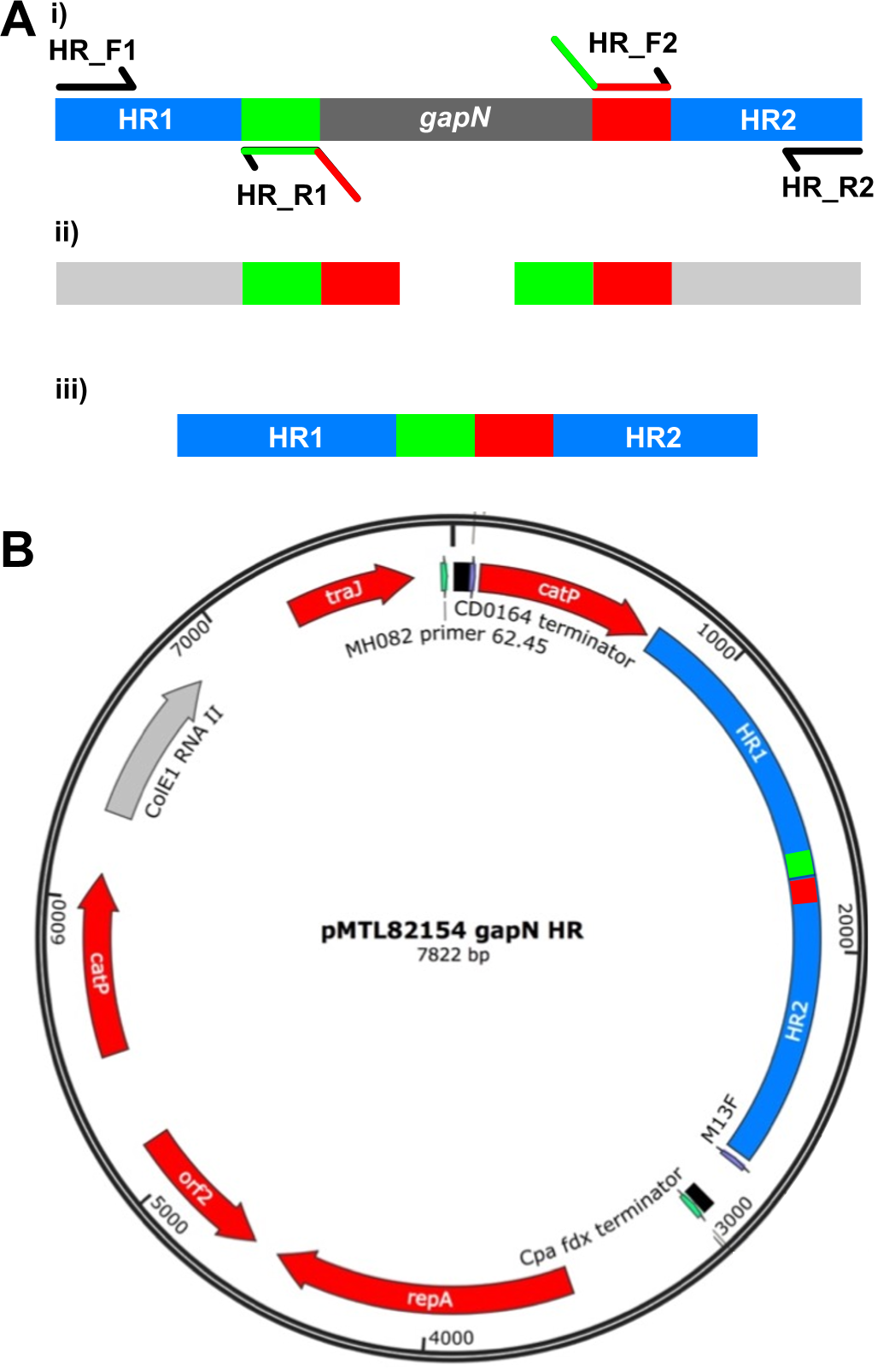

Supplement: Supplementary file 1 — Fig. S1. Generation of a homologous recombination vector for gapN deletion. A) PCR approach to generate the deletion cassette: (i) Amplification of 1 kb fragments upstream and downstream of the gapN gene with 48 bp of complementary sequences; (ii) Two 1 kb PCR products with complementary ends. (iii) Product of overlap‐extension PCR, ready to be blunt‐end ligated into pMTL82154. B) Vector map of ‘pMTL82154_gapN_HR’ that contains the homologous recombination (HR) fragment (i.e. the deletion cassette) cloned into the StuI site of pMTL82154 (verified via StuI restriction digests and sequencing). pMTL82154_gapN_HR contains a pBP1 Gram‐positive replicon, catP antibiotic maker, ColE1 +tra Gram‐negative replicon and a catP reporter gene. [file MBT2-15-1574-s002.tif]

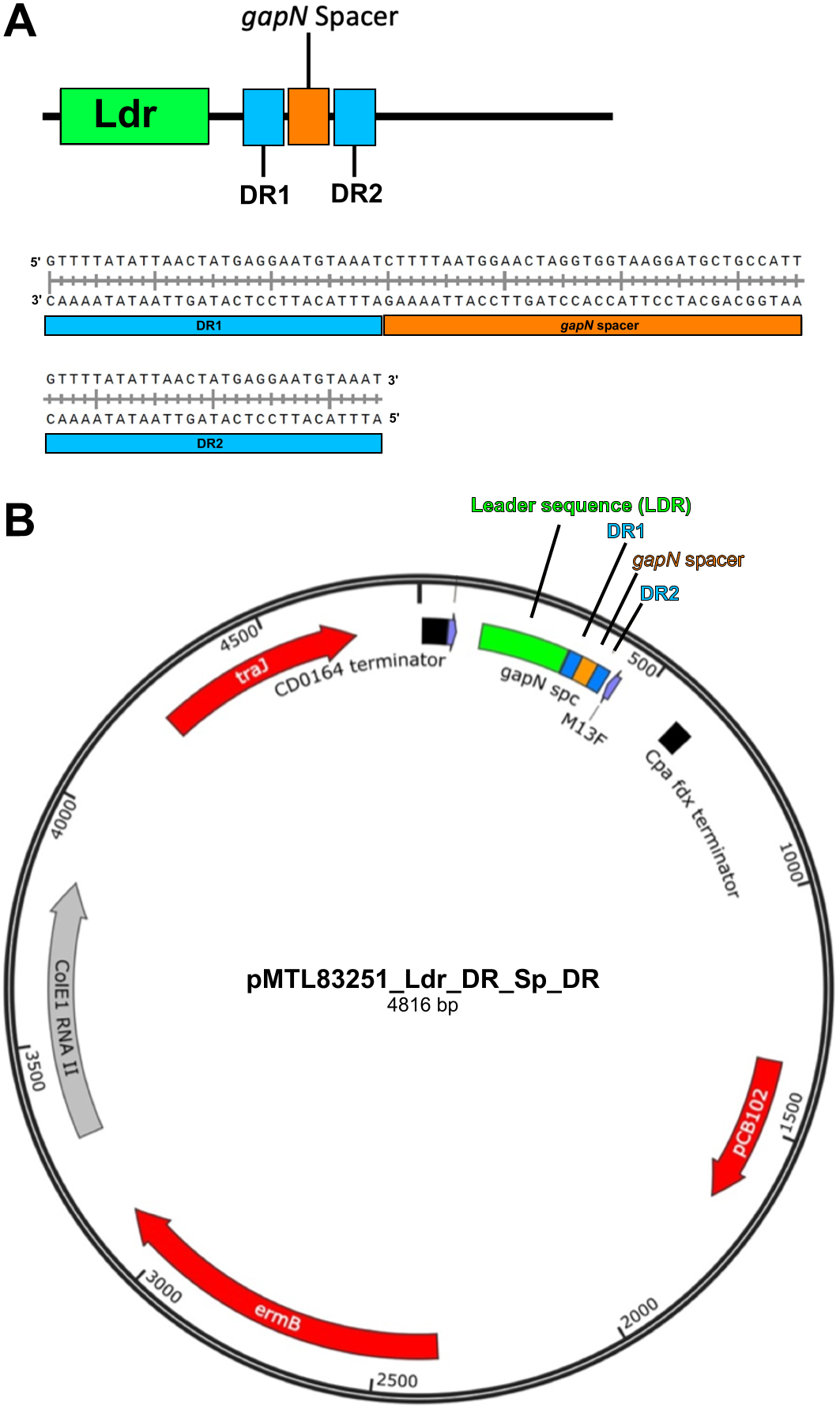

Supplement: Supplementary file 2 — Fig S2. Generation of a killing vector for elimination of transformants that do not contain the gapN deletion. A) Overview of the killing vector targeting cassette for endogenous CRISPR‐Cas for genome editing. The native leader sequence (Ldr) is a 181 bp sequence found downstream of the Cas2 machinery in C. saccharoperbutylacetonicum N1‐4(HMT) (Atmadjaja et al., 2019). The CRISPR/Cas targeting system is comprised of a target‐specific spacer (i.e. gapN spacer) flanked by direct repeats (DR_Sp_DR) that is downstream of the Cas2 sequence. B) Vector map of ‘pMTL83251_Ldr_HR_Sp_HR’ that contains the targeting cassette from panel A. Successful cloning was confirmed via colony PCR and sequencing. [file MBT2-15-1574-s004.tif]

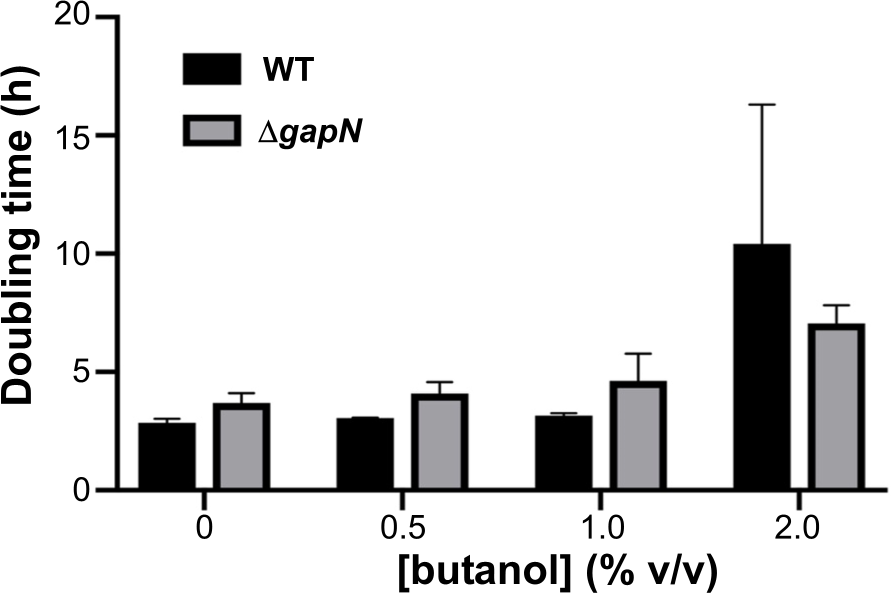

Supplement: Supplementary file 3 — Fig. S3. Butanol toxicity test of wild type (black bars) and ∆gapN (grey bars) strains of C. saccharoperbutylacetonicum N1‐4(HMT). Cells were grown to an OD600 of 1 and were then challenged with varying [butanol]. Doubling times were calculated for the 48 h of growth that followed solvent addition. [file MBT2-15-1574-s001.tif]
