# Supplementary material for: Deletion of glyceraldehyde‐3‐phosphate dehydrogenase (gapN) in Clostridium saccharoperbutylacetonicum N1‐4(HMT) using CLEAVE™ increases the ATP pool and accelerates solvent production
Source: Microb Biotechnol. 2021 Dec 19;15(5):1574–85. doi: 10.1111/1751-7915.13990 (PMC9049615; doi:10.1111/1751-7915.13990)
Supplement: Supplementary file 4 — Table S1. Oligonucleotides used in this study. Table S2. Ratio of nucleotide cofactors in wild type (WT) and ∆gapN C. saccharoperbutylacetonicum N1‐4(HMT). [file MBT2-15-1574-s003.docx]

**Supporting Information**

**Table S1.** Oligonucleotides used in this study.

| Name | Sequence (5′ - 3′) | Description of Function |
| --- | --- | --- |
|  |  |  |
| HR_F1 | ATAATGCTTGCTATTCTAGG | Forward primer used in the amplification of HR1 fragment in deletion cassette |
| HR_R1 | ATCTTACTATTAATTTGTAATAGTTGCTTTATTTTCACCTCTATCCATTG | Reverse primer used in the amplification of HR1 fragment in deletion cassette |
| HR_F2 | AACAATGGATAGAGGTGAAAATAAAGCAACTATTACAAATTAATAGTAAG | Forward primer used in the amplification of HR2 fragment in deletion cassette |
| HR_R2 | AGCTACTTCCATATCAGAAAATTG | Reverse primer used in the amplification of HR2 fragment in deletion cassette |
| M13F  M13R | TGTAAAACGACGGCCAGT  CAGGAAACAGCTATGAC | M13 Primers were used to amplify regions of interest in the pMTL80k plasmids that fell between M13F and M13R |

**Table S2.** Ratio of nucleotide cofactors in wild type (WT) and ∆*gapN* C. *saccharoperbutylacetonicum* N1-4(HMT).

|  | **[NADH]/[NAD^+^]** | | | **[NADPH]/[NADP^+^]** | | | **[NADH]/[NADPH]** | | | |
| --- | --- | --- | --- | --- | --- | --- | --- | --- | --- | --- |
|  | | **WT** | ***∆gapN*** | | **WT** | ***∆gapN*** | | **WT** | ***∆gapN*** |  |
| **4 h** | | 0.48 | 0.84 | | 1.7 | 1.78 | | 5.13 | 7.30 |  |
| **24 h** | | 0.29 | 0.48 | | 0.84 | 0.70 | | 8.81 | 19.52 |  |

**Supporting Information**

**
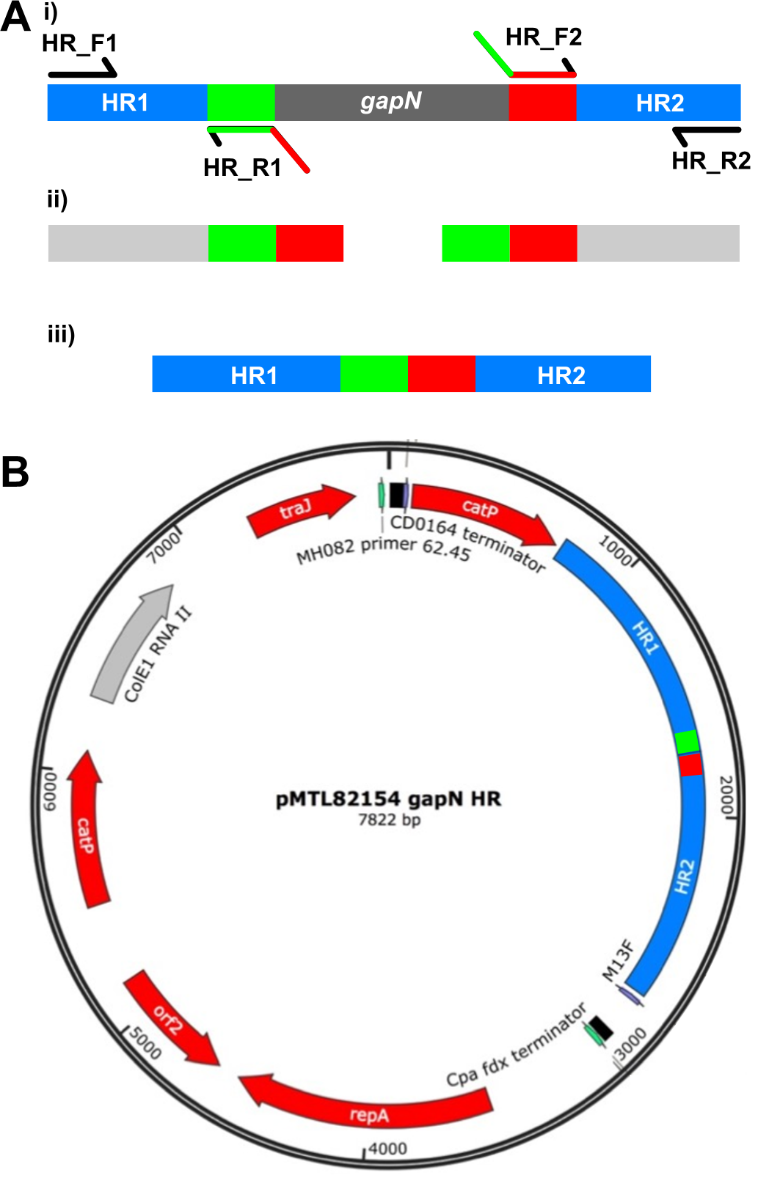
**

Fig. S1. Generation of a homologous recombination vector for *gapN* deletion. A) PCR approach to generate the deletion cassette: i) Amplification of 1 kb fragments upstream and downstream of the *gapN* gene with 48 bp of complementary sequences; ii) Two 1 kb PCR products with complementary ends. iii) Product of overlap-extension PCR, ready to be blunt-end ligated into pMTL82154. B) Vector map of ‘pMTL82154_*gapN*_HR’ that contains the homologous recombination (HR) fragment (i.e. the deletion cassette) cloned into the StuI site of pMTL82154 (verified via StuI restriction digests and sequencing). pMTL82154_*gapN*_HR contains a pBP1 Gram-positive replicon, *catP* antibiotic maker, ColE1 +tra Gram-negative replicon and a *catP* reporter gene.


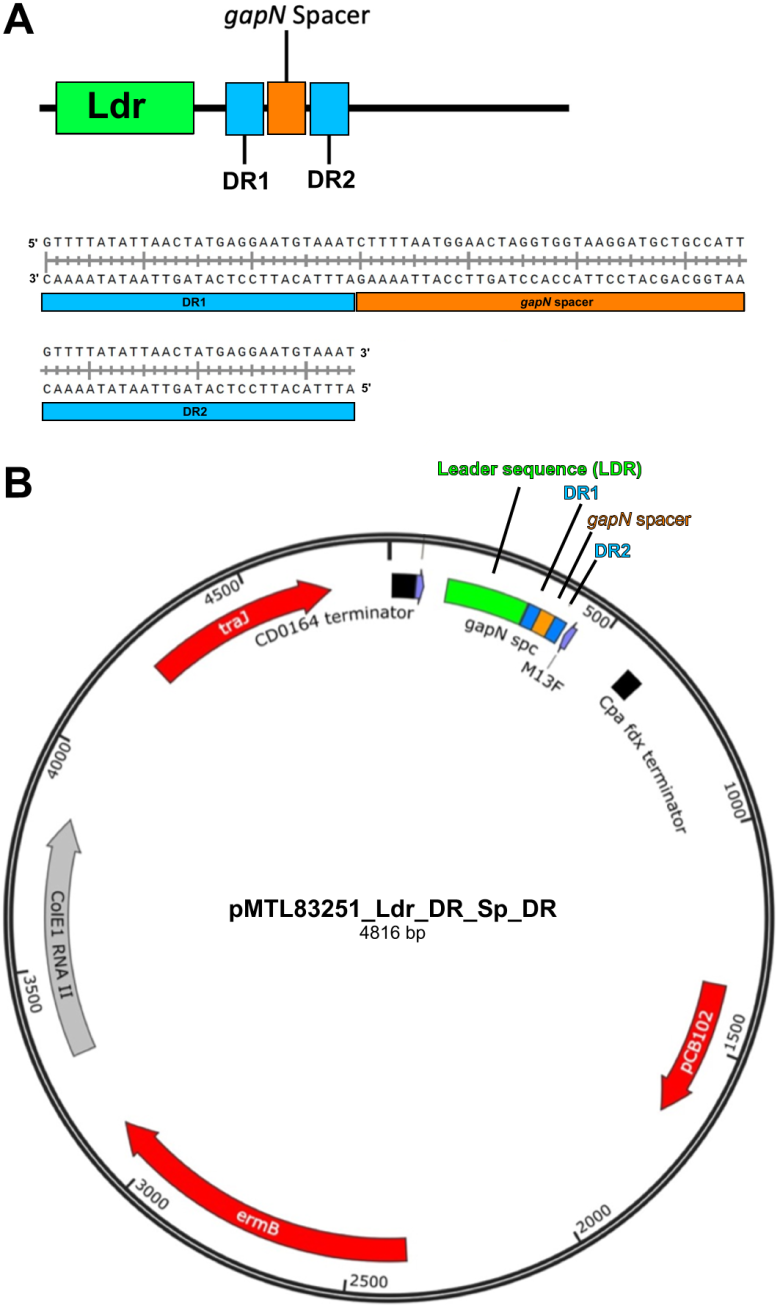


Fig. S2. Generation of a killing vector for elimination of transformants that do not contain the *gapN* deletion. A) Overview of the killing vector targeting cassette for endogenous CRISPR-Cas for genome editing. The native leader sequence (Ldr) is a 181 bp sequence found downstream of the Cas2 machinery in *C. saccharoperbutylacetonicum* N1-4(HMT) (Atmadjaja et al., 2019). The CRISPR/Cas targeting system is comprised of a target-specific spacer (i.e. *gapN* spacer) flanked by direct repeats (DR_Sp_DR) that is downstream of the Cas2 sequence. B) Vector map of ‘pMTL83251_*L*dr_HR_Sp_HR’ that contains the targeting cassette from panel A. Successful cloning was confirmed via colony PCR and sequencing.


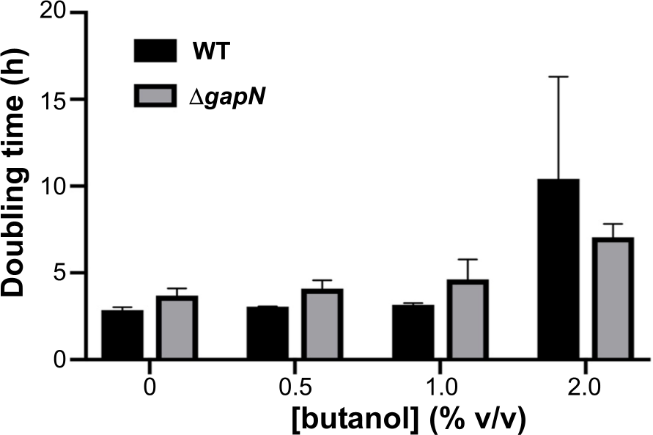


Fig. S3. Butanol toxicity test of wild type (black bars) and ∆*gapN* (grey bars) strains of *C. saccharoperbutylacetonicum* N1-4(HMT). Cells were grown to an OD_600_ of 1 and were then challenged with varying [butanol]. Doubling times were calculated for the 48 h of growth that followed solvent addition.
